# Current Status and Challenges of Stem Cell Treatment for Alzheimer’s Disease

**DOI:** 10.3233/JAD-200863

**Published:** 2021-11-23

**Authors:** Mar Pacheco-Herrero, Luis O. Soto-Rojas, Heidy Reyes-Sabater, Linda Garcés-Ramirez, Fidel de la Cruz López, Ignacio Villanueva-Fierro, José Luna-Muñoz

**Affiliations:** a Neuroscience Research Laboratory, Faculty of Health Sciences, Pontificia Universidad Católica Madre y Maestra, Dominican Republic; b Facultad de Estudios Superiores Iztacala, Universidad Nacional Autónoma de México, State of Mexico, Mexico; c Escuela Nacional de Ciencias Biológicas, Depto de Fisiología, Instituto Politécnico Nacional, Mexico City, Mexico; d Instituto Politécnico Nacional, CIIDIR, Unidad Durango, Durango, México; e National Dementia BioBank, Ciencias Biológicas, Facultad de Estudios Superiores Cuautitlán, UNAM, State of Mexico, Mexico; f Banco Nacional de Cerebros-UNPHU, Universidad Nacional Pedro Henríquez Ureña, Dominican Republic

**Keywords:** Alzheimer’s disease, amyloid-β, neural stem cells, neurodegeneration, stem cells, tau protein, therapy

## Abstract

Neurodegenerative diseases called tauopathies, such as Alzheimer’s disease (AD), frontotemporal dementia, progressive supranuclear palsy, and Parkinson’s disease, among others, are characterized by the pathological processing and accumulation of tau protein. AD is the most prevalent neurodegenerative disease and is characterized by two lesions: neurofibrillary tangles (NFTs) and neuritic plaques. The presence of NFTs in the hippocampus and neocortex in early and advanced stages, respectively, correlates with the patient’s cognitive deterioration. So far, no drugs can prevent, decrease, or limit neuronal death due to abnormal pathological tau accumulation. Among potential non-pharmacological treatments, physical exercise has been shown to stimulate the development of stem cells (SCs) and may be useful in early stages. However, this does not prevent neuronal death from the massive accumulation of NFTs. In recent years, SCs therapies have emerged as a promising tool to repopulate areas involved in cognition in neurodegenerative diseases. Unfortunately, protocols for SCs therapy are still being developed and the mechanism of action of such therapy remains unclear. In this review, we show the advances and limitations of SCs therapy. Finally, we provide a critical analysis of its clinical use for AD.

## INTRODUCTION

**Fig. 1:**
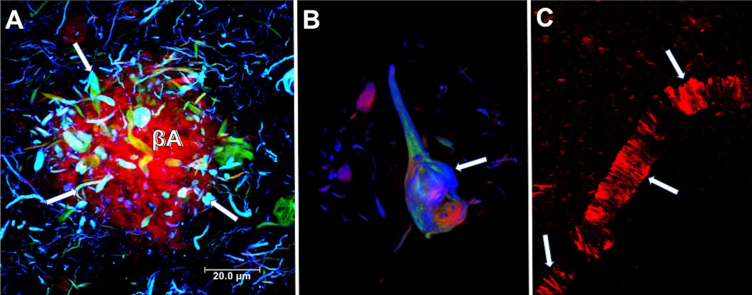
Neuropathological lesions in the brain from a case with AD. A) Neuritic plaque. Deposits of extracellular amyloid-β (Aβ), lined with dystrophic neurites (arrows). B) Intracellular neurofibrillary tangle. Different processing stages of the tau protein are shown in the neuronal soma (arrow). A and B were immunostained using two tau antibodies, one directed against the phosphorylated pT231 site (green channel) and TG-3 (blue channel). Sections were counterstained with thiazine red dye. C) Blood vessel with fibrillar amyloid angiopathy stained by the thiazine red dye (arrows). Thiazine red is a red fluorescent dye (560 nm) with an affinity for beta-amyloid peptide and fibrillar tau protein.

It has been estimated that over 50 million people live with dementia globally, a number set to increase to 152 million by 2050. Alzheimer’s disease (AD) is the most common cause of dementia, representing an estimated 50–60% of cases. It is most common in individuals over 70 years, with prevalence increasing with age [1]. In the majority of cases, AD is sporadic, with no clear etiology. Less than 5% of cases have a genetic origin, transmitting in an autosomal dominant way across multiple generations [2]. AD is a neurodegenerative disease marked by irreversible neuronal death resulting in progressive and disabling impairment of cognitive functions, such as memory, language, reasoning, behavior, and executive function, which interfere with the essential daily activity of the person. AD brain neuropathology is characterized classically by two hallmark lesions: neuritic plaques (NPs, Fig. 1A) constituted by extracellular deposits of the amyloid-β peptide (Aβ), and neurofibrillary tangles (NFTs, Fig. 1B) composed of paired helical filaments (PHFs), whose main constituent is tau protein. Synaptic loss in AD brains has been suggested to be best correlated with cognitive decline [3]. The collapse of neural networks, including the death of neurons and degeneration of synapses, could be caused by the accumulation of toxic soluble forms of Aβ and tau at the synapse [4]. Aβ protein is derived from the amyloid-β protein precursor (AβPP). AβPP is cleaved by *α*-secretase within the Aβ domain, avoiding the generation of Aβ in the non-amyloidogenic pathway. On the other hand, AβPP can be sequentially cleaved by the β- and *γ*-secretases at the N- and C-terminus of the Aβ domain, respectively, generating Aβ in the amyloidogenic pathway [5]. The Aβ production, which is thought to be produced primarily by neuronal cells, is not inherently toxic and might even have a physiological function [6, 7]. However, it has been widely demonstrated that Aβ has a toxic role when it accumulates in the neuronal parenchyma as NPs or cerebral and leptomeningeal blood vessels as cerebral amyloid angiopathy (Fig. 1C) [8]. All forms of amyloid plaques, including diffuse plaques, are associated with neuropathology, mainly characterized by neuritic and synaptic dystrophies. NPs are composed of a multitude of highly aggregated Aβ fibrils. It has been seen that shortened/modified Aβ forms are significantly more resistant to degradation and aggregate more rapidly. Aβ_40_ (with 40 amino acid residues) and Aβ_42_ (with 42 residues) are the major variants involved in AD pathology [6, 7]. AβN3 (pE), a peptide bearing amino-terminal pyroglutamate at position 3, has been demonstrated to be a significant N-truncated/modified constituent of Aβ deposits [9–11]. AβN3 (pE) accumulates in the brain at the earliest stages of AD, suggesting that it may lead to pathological amyloid aggregates [11]. The blood–brain barrier (BBB) is a complex structure composed of endothelial cells, pericytes, and astrocytic end-feet. Since the BBB is crucial for the Aβ clearance from the brain, neurovascular dysfunction (Fig. 1C) may play a critical role in AD development [12]. On the other hand, tau is an intracellular microtubule-associated protein, essential for structural support and axonal transport.

**Fig. 2:**
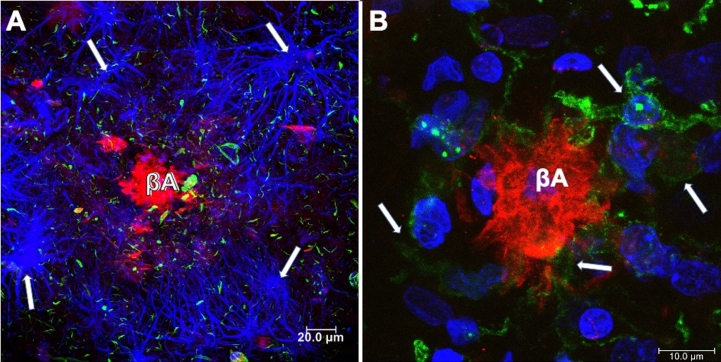
Neuritic plaques (NPs) in the brain from a case with AD. A) NPs surrounded by glial cells (anti GFAP-blue channel). Aβ was revealed using Thiazine red (red channel) and tau with anti pT231 (green channel). Dystrophic neurites associated with amyloid plaque and throughout the neuropil were observed. B) Microglial cells (anti-Iba-1; green channel) and fibrillar Aβ deposit (red channel; Thiazine red), counterstained with To-Pro Iodide (blue channel; nuclei).

It becomes abnormally hyperphosphorylated and truncated [13, 14], giving rise to the formation of PHFs and NFTs within the cytoplasm of vulnerable cells [15, 16]. The presence of astrocytes (Fig. 2A) and reactive microglia (Fig. 2B) surrounding and within Aβ plaques, respectively, suggest that the neuroinflammatory response may increase cognitive decline during AD progression [17, 18]. Astrocytes may play an additional role in AD by secreting significant quantities of Aβ, raising levels of AβPP, and β- and *γ*-secretases [19], and contributing to Aβ clearance [20]. The currently approved AD treatments are largely symptomatic, being unable to prevent disease progression. These treatments include cholinesterase inhibitors for patients with any stage of AD dementia and N-methyl D-aspartate (NMDA) antagonist, or memantine, to treat moderate to severe AD, or a combination of both [21]. Anti-Aβ and tau immunotherapies are being tested, but with limited success to date. Aβ accumulation appears to be more of a physiological compensatory mechanism than the pathological initiator [22]. In the case of tau, the antibodies should bind to the aggregated forms generated intracellularly or prevent tau seeding and spreading caused by aberrant tau without impacting its physiological function of tau [23–25]. In recent years, stem cell (SCs) therapies have emerged as a putative biological tool to treat a range of diseases without successful treatment. This review focuses on analyzing SC therapy with a critical vision for its clinical use in AD.

## TYPES OF STEM CELLS: CHARACTERISTICS, BENEFITS, AND LIMITATIONS

SCs are undifferentiated cells that have the ability of self–renewal to replicate rapidly and continuously, as well as giving rise to new SCs and specialized cells under appropriate conditions. Asymmetric mitosis is the process that allows a SC to obtain two intrinsically different daughter cells. Cell fate-determining factors are provided extrinsically or inherently to SCs in a polarized manner. By coordinating the cell axis polarity, the daughters of the SCs acquire distinct fates: self-renew or commit to differentiation [26, 27]. The types of SCs vary according to their differentiation potential [28], which reduces at each step. This means that a unipotent SC cannot differentiate into as many cell types as a pluripotent SC. The zygotes are totipotent cells, the most developmentally expansive cells. Yet they are rarely considered an SCs in mammals because they cleave into blastomeres of equal developmental potency for at most three cell divisions, and therefore manifest minimal self-renewal potential [29]. Below, we will describe and discuss the characteristics and the advantages and disadvantages of the three main types of SCs.

### Embryonic stem cells

Embryonic SCs (ESCs) are pluripotent SCs derived from the inner cell mass of a blastocyst [30]. They have the advantage of being pluripotent and theoretically can origin any type of cell (being exposed to the appropriate stimuli). Therefore, they present a greater therapeutic range. However, there are important limitations to implement therapeutic strategies from ESCs: a) difficulty in directing and differentiating them in a particular cell type; b) possibility of turning into cancerous tissue; c) once transplanted, an immune response may occur leading to their rejection by the host; d) ethical considerations for collecting cells from a viable embryo [31].

### Induced pluripotent stem cells

Induced pluripotent stem cells (iPSCs) are a type of pluripotent SCs that can be generated directly from adult SCs (see adult stem cells, below). Therefore, iPSCs have been reprogrammed back into an embryonic-like pluripotent state. iPSCs can be directed to differentiate into the most suitable cell type and model the events of a specific disease, having the benefit of presenting a diverse therapeutic spectrum. For example, dermal fibroblasts have differentiated directly into dopaminergic neurons through viral co-transduction of forebrain transcriptional regulators in media, which promote neuronal survival [32]. iPSC-based technology has also been beneficial for creating *in vitro* models of various neurodegenerative diseases such as Parkinson’s disease, Huntington’s disease, amyotrophic lateral sclerosis, and AD [33, 34]. In addition, it can be useful to study the underlying pathophysiological mechanisms of these and more disorders.

### Adult stem cells

**Fig. 3:**
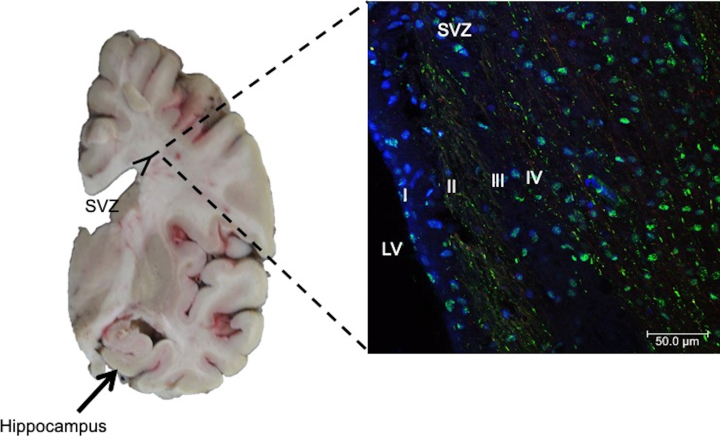
Left. Coronal section of human brain. Neural stem cells in the subgranular zone of the hippocampal dentate gyrus (arrows) and subventricular zone (SVZ), lining the walls of the lateral ventricles (LV), have been reported. Right. Immunofluorescent staining of SVZ in AD brain. Antibody against phospho-tau protein and counterstaining with To-Pro Iodide was used to reveal nuclei. Four layers in the SVZ are observed: I) ependymal layer, II) hypocellular gap, III) astrocytic ribbon layer, and IV) transitional zone. Staining for phospho-tau protein showed dense labeling of speckles associated with nuclei.

Adult SCs are also called tissue-specific SCs, because they are lineage-restricted cells and generally habit inside “niches” in their tissue of origin. Adult SCs can only differentiate into a specific mature cell of the organ in which they reside, with characteristic morphologies and specialized functions [35]. Adult SCs are mainly found in bone marrow, skin, muscles, and intestine [35–38]. These SCs are inactive, but their replication and differentiation can be induced after an injury and thus replace the dead cells. The mechanisms for this event to occur are unknown. Several therapies based on adult SCs have been developed. Nevertheless, few of these have been approved. The main types are: 1) Hematopoietic stem cells (HSCs) that have been used to repopulate the bone marrow in patients with hematological disorders [39–41]. HSCs can also be collected at birth from umbilical cord blood [42] and can only be used to try to reconstitute the hematopoietic system [43, 44]. 2) Mesenchymal stem cells (MSCs), a subset of adult SCs that have several therapeutic advantages: a) they are abundant and readily available in a variety of mesenchymal tissues [45–47]; b) they can differentiate into several cell types such as adipocytes, osteoblasts, and chondrocytes [48] and, for this reason, they can have therapeutic implications in more pathological entities compared to other SCs types; c) they have potent paracrine effects [49, 50], which implies that an injured tissue could have the ability to repair itself. Both MSCs and HSCs do not necessarily require reprogramming for their therapeutical application. 3) Neural stem cells (NSCs), which are also multipotent, are responsible for generating all neural cell types during development: neurons, astrocytes, and oligodendrocytes. Depending on the developmental stage and location within the central nervous system (CNS), NSCs may generate neurons, astrocytes, or oligodendrocytes. In the adult brain, NSCs niche comprises the subventricular zone (SVZ) and the subgranular zone (SGZ) of the dentate gyrus in the hippocampus and the external germinal layer of the cerebellum [51, 52] (Fig. 3). Under physiological conditions, neurogenesis in the adult hippocampus is related to cognitive processes such as memory and learning. Therefore, its dysregulation could be associated with cognitive impairment as occurs in AD. However, the molecular mechanisms involved in human neurogenesis are still unclear since most studies have been carried out in rodent models and postmortem human brain tissue [53]. Interestingly, in addition to SVZ and SGZ, the hypothalamus has been suggested as a neurogenic niche in the CNS. *α*-Tanycytes have been proposed to give rise to the hypothalamic SCs population. Hypothalamic neurogenesis plays a vital role in controlling and regulating energy metabolism and feeding [54].

## CONSIDERATIONS FOR STEM CELL THERAPY

SCs therapy includes several critical stages, each of which must be taken into account.

### Collection of donor cells

There are two main methods for obtaining SCs: a) pre-implanted embryos of 4 days after fertilization occurs to extract ESCs or b) induce adult SCs that have already been differentiated into a pluripotent state [55, 56]. The latter can be obtained from adult tissues or organs; and hence they are relatively accessible but have limited capacity since they can only differentiate into a particular type of cells. The most used are bone marrow, muscle tissue, fat tissue, periodontal ligament, peripheral blood, synovial fluid, salivary gland, alveolar epithelium, umbilical cord, and dental pulp [57]. To the iPSCs, viral transfection of a group of crucial genes: oct4, sox2, xmyc, and klf4, into somatic cells are usually used [57]. In a recent systematic multi-database analysis regarding pluripotent SCs, it was determined that most clinical trials involved iPSCs (74.8%) versus ESCs (25.2%). However, in interventional studies, the majority used ESCs (73.3%).

### Storage of stem cells

SCs banking is used to collect, store, and preserve SCs for research. These cells are kept in cell cultures, which need to be strictly controlled in terms of pH, temperature, humidity, and gas composition. Other factors, such as the authenticity of the lineage of SCs, lack of cross-contamination, absence of microbiological contamination, and correct maintenance of the stability and integrity *in vitro*, should be periodically monitored [58]. Each step has to be optimized and scrutinized to ensure that cells are retained in an undifferentiated stage. An excellent cryopreservation method depends on optimal freezing, controlled frozen storage, and proper thawing techniques [59, 60]. An incorrect thawing process can affect the viability and function of SCs, with rapid thawing in a bath at 37°C or a higher temperature being recommended [59]. It is also necessary to have a washing process to remove the cryopreservative, which can be considered toxic [61].

### Harvesting and differentiation of stem cells

The further an SC is differentiated, the more specialized it becomes. It is believed that the differentiation process is defined by internal signals controlled by cellular genes and by external signals that include chemicals secreted by other cells, physical contact with neighboring cells, and the specific molecular microenvironment. The interaction of signals and cellular DNA makes the cell acquire epigenetic marks that restrict the DNA expression, and these can be transmitted through cell division. Manipulating the culture conditions, therefore, are essential for the *in vitro* harvesting and differentiation of cells. In general, inhibitory factors such as transcription factors are used to block differentiation processes: OCT3-4, NANOG, and SOX2. For differentiation to a specific cell type, it is essential to understand the signaling pathways to select the correct choice of differentiation factors [55, 56]. A protein called nestin is expressed in all precursor cells throughout the embryonic nervous system, giving rise to neurons or glia. Nestin expression is turned off abruptly in the nervous system when the cells become postmitotic and functionally committed to becoming neurons or glia [62]. Nestin is expressed in cell cultures for a long time. For example, Fraichard and colleagues induced mouse ESCs to differentiate into precursors of neurons and glial cells with retinoic acid. These cells were identified three days after the onset of differentiation [63]. In another study, placental-derived cells were also driven to differentiate into neural cells with human brain-derived neurotrophic factor (hBDNF) and retinoic acid [45]. Fibroblast growth factor-2, epidermal growth factor, Sonic hedgehog, fibroblast growth factor-8, and bone morphogenetic protein-4 induce neuronal or glial differentiation in tissue culture [64]. The European Registry of Embryonic Stem Cell Lines is one of the most potent registers that gather information on cell lines and their form of culture. However, changes in cell proliferation (density), variation in the concentration of factors present in the environment, tight control over the confluence of the culture, and generation of cell-cell and cell-matrix junctions, should all be taken into account. The efficacy of differentiation factors in culture media depends on functional maturity, efficiency, and introducing cells into an environment equivalent to that found *in vivo*. Topography, shear stress, and substrate rigidity influence the phenotype of future cells [65]. *In vivo*, once the SCs have been transplanted, the interrelationship between them and the microenvironment in which they have been placed is impossible to control precisely. The possibility that the cell lineage changes has to be considered. The “plasticity” or ability of one type of SCs to undergo a transition to a cell from other lineages has been reported [64, 66, 67]. For example, Jackson and colleagues discovered that a group of muscle cells could become blood cells and that they were later dealing with a subpopulation of cells that typically reside in muscle tissue [68]. Similarly, the SCs must integrate and behave as a structural and functional unit with the rest of the cells: within the brain, the SCs have to generate electrical impulses and release and respond to chemicals. Allowing for all of these complex requirements, restoration of affected brain sites should still be possible.

### Stem cell viability and transplantation

Cell viability and growth status can be quantified by counting cell doubling time, telomerase activity, cell cycle, and cloning efficiency. The trypan blue exclusion test is often used to determine the viability of thawed SCs, a method in which viable cells fail to take up blue dye because of their intact cell membranes. There are many more viability assays according to the characteristics of specific SCs, like 7-aminoactinomycin and propidium iodide, among others. The World Health Organization (WHO) recommends that the viability of thawed SCs, from a bank should be 80% or greater, depending on the cell lineage, although 70% may still be acceptable [58]. Meanwhile, for somatic cellular therapies, the minimum acceptable viability specification recommended by the U.S. Food and Drug Administration (FDA) is generally set at 70% [69]. Stem cell transplantation is a procedure in which a patient receives healthy SCs to replace damaged SCs. The main types of stem cell transplantation are (a) autologous transplantation, where the patient’s own SCs are used to avoid compatibility issues and (b) allogeneic transplantation, where the SC comes from a donor who may be a family member or someone unrelated to the patient [55]. Whether the administration of SCs should be peripheral or local, has yet to be defined. At Kyoto University Hospital, Takayuki Kikuchi deposited dopamine precursor cells into the brain of a patient with Parkinson’s disease at 12 sites, known to be centers of dopamine activity, after research with a primate model of Parkinson’s disease [70]. Although researchers reported that the patient had no adverse reactions to the cells in the short term, no further details of its efficacy and long-term safety have been disclosed. In the case of AD, most clinical trials perform several stereotactic injections in the hippocampus, precuneus, or other regions (see clinical trials in the next section). How these cells migrate from injection sites to other affected areas of the brain remains elusive [71, 72].

### Control of stem cell proliferation and effectiveness

*In vitro*, to prevent the possibility that human SCs might develop into embryogenic structures with the potential to become a living organism, the International Society for Stem Cell Research (ISSCR) states that these should be kept in culture just for the minimum period necessary for the study. The “14-day rule” [73] restricts the cultivation of human embryos to the 14th-day post-fertilization [74]. *In vivo*, one of the main concerns is the ability to monitor non-invasively the transplanted cells in the same individuals over time, from new localization to viability, migration, and differentiation. There are two main classes of cell-labeling methods: direct and indirect. For direct, a labeling agent is introduced into the cell before transplantation, mainly radiotracers, nanoparticles, or quantum dots. The disadvantage of this method is that it only allows short-term tracing because of the decreasing imaging signal [75–78]. For the indirect labeling method, a reporter gene transduced into the cell before transplantation is visualized upon the injection of a specific probe. This method offers the advantage of long-term imaging for cell survival and longitudinal measurements. Also, it generates signals dependent on cell viability [79]. However, disadvantages include the cost, cellular dysfunction, cell death, immunogenicity of the gene product, and potential risk for uncontrolled growth or malignancy [75]. Bioluminescence imaging, fluorescence imaging, single-photon emission computed tomography, magnetic resonance imaging, or positron emission tomography are some of the imaging systems used in conjunction with these technologies [79–81].

## STEM CELL THERAPY IN ALZHEIMER’S DISEASE

To date, the mechanism of action by which SCs therapy may exert its effects remains unclear. Also, it is necessary to conduct several studies that prove the safety and efficacy of AD treatment. It is important to emphasize that in addition to cell replacement therapy, SCs therapy could have neuroprotective effects through antioxidant, anti-inflammatory, antiapoptotic, angiogenic, and neurogenic mechanisms [82, 83].

Several preclinical studies for AD have provided promising results. It has been proposed that SC therapy may have effects by reducing amyloid plaques, hyperphosphorylation of tau [84], and a decrease neuroinflammation environment, consistent with the observations from murine AD models [85]. Likewise, SC therapy could have immunomodulatory effect, regulating the activity of pro-inflammatory cytokines [86] and vascular endothelial growth factor [87]. Also, this therapy could favor the neurogenesis and synaptogenesis process and improve the cognitive deficits [86, 88, 89]. These effects can be obtained by regulating metabolic activity, the anti-inflammatory factors secretion, and various signaling pathways associated with the BDNF and nerve growth factor [90, 91].

In this sense, hundreds of experiments have been carried out in animal models [90, 91]. Although some studies have shown encouraging results, some aspects have not been sufficiently demonstrated. Marsh and colleagues analyzed the long-term effect of human NSCs transplantation in an immune-deficient mouse model of AD (Rag-5xFAD mice). Five months after transplantation, they found no evidence of improved learning and memory, no effect on amyloid levels, no changes in brain-derived neurotrophic factor, no glial or neuronal differentiation, and no increase in synaptic density [92]. In the mice 3xTg-AD model, in contrast with what occurs in AD patients, there is no massive neuronal death in the hippocampus due to the intracellular accumulation of NFTs. This is because there is an expression of phosphorylated tau protein [93] but not of truncated tau, which favors polymerization into PHFs [15, 94–97]. Thus, after repopulation with human NSCs of areas such as the hippocampus and cortex, where there is no massive neuronal death in this model, no changes in synaptic density or amyloid deposits would be expected. Most likely, the unfavorable results in these types of studies are due to the use of human SCs in mice. The correct experimental design would imply the use of SCs of the same species. Another important aspect is that the time frame for the efficacy of the transplanted SCs over time. The optimal time frame for AD is not clear. In one study, NSCs were transplanted into the brains of TG2576 mice at 12- and 15-months of age brains. Although memory in the 12-month-old mice improved, this was not the case for the 15-month-old mice [98]. Importantly, these studies require that the implanted SCs be labeled to monitor their migration to the affected brain areas. MSCs in young mice were found in the lung, axillary lymph nodes, blood, kidney, bone marrow, spleen, liver, heart, and brain cortex. In contrast, young MSCs that were transplanted into aged mice were found only in the brain cortex. In both young and aged mouse recipients, transplantation of aged MSCs showed biodistribution only in the blood and spleen [99].

The concentration of cells delivered also significantly influences the distribution, viability, and efficacy of the method. Kim et al. observed that, at three different concentrations delivered intracerebroventricularly, MSCs were more widespread and viable using the lower concentration. This suggests that a lower dose could have a therapeutic action by releasing paracrine factors [100]. Aspects like the mechanism of stimulation of the neurogenic niche, or the relationship between transplanted MSCs in the ventricular area and the cerebrospinal fluid, are not clear. Another concern of the transplantation of SCs is the appearance of adverse effects. In one study, neural SCs (253G1-NSs) were transplanted to treat spinal cord injury in mice, and the long-term safety efficacy was assessed. Although the mice experienced an improvement of motor function up to 47 days after transplantation, there was a gradual deterioration in motor function, followed by the proliferation of grafted cells and tumor development. These findings suggest that cell proliferation and induced tumor formation increase over time [101]. Despite the results with animal models, most registered clinical trials using SCs in humans are at an early stage, where their safety and efficacy have yet to be validated and any treatments approved by regulatory agencies. To date, there are only a few registered clinical trials using cellular therapy for AD that have been completed. The results of other studies have yet to be reported or, in some cases, have been suspended (Clinicaltrials.gov. https://clinicaltrials.gov Accessed June 2021) (Table 1). Of the completed trials, NCT03117738 tested the efficacy of autologous AdMSCs administrated intravenously. The procedure was repeated nine times at 2-week intervals. Although no study results have been reported, a follow-up clinical study compares the efficacy and safety of these SCs versus donepezil (NCT04482413). In NCT01297218, the safety and effectiveness of stereotactic brain injection of human umbilical cord blood-derived mesenchymal stem cells (hUCB-MSCs) were tested in mild to moderate AD. Nine patients with AD received treatment and were followed for 12 weeks and up to 24 months. There was no dose-limiting toxicity. The administration of hUCB-MSCs into the hippocampus and precuneus by stereotactic injection did not produce any serious adverse events, just some minor side effects such as wound pain, headache, dizziness, and delirium. It is difficult to understand how an older adult can withstand surgery of this nature and the needle damage in the injection. Although hUCB-MSCs administration for 40 days into the hippocampus of 10-month-old APPswe/PS1dE9 mice decreased Aβ_42_ levels and plaque formation [102], this effect could not be replicated in AD patients [103]. We suggest that applying the SCs inoculum in layer two of the entorhinal cortex (EC), instead of the hippocampus, would be more appropriate. This is because EC is one of the first areas affected in AD and most sensitive to neuronal death due to the accumulation of NFTs [104–106]. How neuronal differentiation is induced in these areas after transplantation needs to be clarified. Besides, in this clinical trial, authors reported that the rate of cognitive decline in the patients was faster (nine-point drop in Mini-Mental State Examination (MMSE) score within two years) following this intervention, than typical AD progression (three-point drop in MMSE score per year) [98]. The investigators argued that different brain pathologic environments might explain the different responses to MSCs treatment. Also, the early-onset AD of most participants could justify the rapid progression in cognitive decline after the therapy. The lack of healthy control participants and a small sample size precluded any conclusion drawn from the study [98]. Other clinical trials completed in the first phase, have initiated a second clinical trial consisting of a long-term follow-up study to obtain safety and efficacy data (for example, NCT02054208 and NCT03172117).

**Table 1:** Clinical trials with stem cells for the treatment of Alzheimer’s disease. The active and complete clinical trials are shown. Information obtained from http://clinicaltrials.gov, until May 27, 2020

Clinical trial no.	Title	Intervention	Status	Country	Sponsor	References
NCT03172117	Follow-up study of safety and efficacy NEUROSTEM clinical trial (NCT02054208)	A long-term follow-up study to obtain safety and efficacy data in subjects who completed phase 1/2a clinical trial of NEUROSTEM (human umbilical cord blood-derived mesenchymal stem cells)	Active (estimated study completion date: August 2022)	Korea	Medipost Co Ltd.	*ClinicalTrials.Gov*
NCT03724136	Alzheimer’s Autism and Cognitive Impairment Stem Cell Treatment Study (ACIST)	Non-randomized, parallel assignment, and clinical trial to evaluate the use of autologous Bone Marrow-Derived Stem Cells (BMSC) to improve cognitive impairment in AD. 100 patients will be assigned to each of the 3 groups: Group 1: Intravenous administration of 14 cc of BMSC fraction. Group 2: Intravenous administration of 14 cc of BMSC fraction combined with Near-Infrared Light exposure. Group 3: Intravenous administration of 14 cc of BMSC fraction combined with Intranasal topical 1 cc of BMSC fraction.	Active (estimated study completion date: October 2022)	United States	MD Stem Cells	*ClinicalTrials.Gov*
NCT04388982	Safety and Efficacy Evaluation of exosomes derived from Allogenic Adipose Mesenchymal stem cells (MSC-Exos) in Patients With Alzheimer’s Disease	Single-center, open-label, phase I/II clinical trial. To explore the safety and efficacy of MSC-Exos in mild to moderate AD. 9 subjects will be assigned to one of the three study groups: low (5*μ*g), mild (10*μ*g), or high (20*μ*g) dosage of MSC-Exos. Administrated for nasal drip twice a week, for 12 weeks.	Active (estimated study completion date: April 2022).	China	Cellular Biomedicine Group Ltd	*ClinicalTrials.Gov*
NCT04040348	Safety, possible side effects, effectiveness of mesenchymal stem cell infusions in mild to moderate AD patients.	Single group assignment, open label, phase I. To evaluate the safety, tolerability, and outcomes of multiple allogeneic human MSC infusions in mild to moderate AD patients. Participants in the treatment group will receive a total of 4 doses. Each dose (100 million cells) will be administered (intravenously) one about 13 weeks within a year period.	Active (estimated study completion date: September 2022)	United States	Bernard (Barry) Baumel	*ClinicalTrials.Gov*
NCT02899091	Evaluation of the Safety and Potential Therapeutic Effects After Intravenous Transplantation of placenta-derived mesenchymal stem cells (CB-AC-02) in Patients with Alzheimer’s Disease	Randomized, Double-blind, Placebo-controlled, Phase I/IIa Clinical Trial to evaluate the safety and the potential therapeutic effects of CB-AC-02 in AD. 24 patients will be randomized in the treatment cohorts: intravenous transplantation of 2.0×10^∧^8 cells on day 0, and 4 weeks later, or placebo.	Active (estimated study completion date: December 2021)	Korea	CHABiotech CO., Ltd	*ClinicalTrials.Gov*
NCT04228666	A Clinical Trial to Determine the Safety and Efficacy of Hope Biosciences autologous adipose-derived mesenchymal Stem Cell Therapy (HB-adMSCs) for the Treatment of Alzheimer’s Disease	Phase I/IIa, open-label, non-randomized study in 24 subjects with AD. Administration of four intravenous infusions of autologous adipose-derived mesenchymal stem cells (HB-adMSC) (2×10^∧^8 total HB-adMSC cells), comparison between baseline data and follow-up to evaluate the safety profile, will be performed.	Active (estimated study completion date: February 2021)	United States	Hope Biosciences	*ClinicalTrials.Gov*
NCT02600130	Allogeneic Human Mesenchymal Stem Cell Infusion Versus Placebo in Patients with Alzheimer’s Disease	Phase I, prospective, randomized, placebo-controlled, double-blinded study. To evaluate the safety and efficacy of Longeveron Mesenchymal Stem Cells (LMSCs) for the treatment of AD. 25 subjects will be randomized to (2 : 2:1) to receive peripheral intravenous infusions of low-dose LMSCs (20×10^∧^6), high-dose LMSCs (100×10^∧^6) or placebo (Plasmalyte A and 1% human serum albumin (HSA)). Subjects will be followed up at 2, 4, 13, 26, 39, and 52-week post-study product infusion.	Active (estimated study completion date: September 2020).	United States	Longeveron LLC	*ClinicalTrials.Gov*
NCT02833792	Allogeneic Human Mesenchymal Stem Cells (hMSCs) for Alzheimer’s Disease	Phase IIa multi-center, randomized, single-blind, placebo-controlled, crossover study in subjects with mild to moderate AD. 2 cohorts of subjects (20 subjects per group), randomized in a 1 : 1 allocation to first receive active study drug (intravenous infusion of 1.5×10^∧^6 hMSCs per kg) or placebo (Lactate ringer’s solution). After six months, each subject will be switched to the other treatment. Efficacy and safety by neurologic, functional, and psychiatric endpoints will be evaluated.	Active (estimated study completion date: June 2020).	United States	Stemedica Cell Technologies, Inc.	*ClinicalTrials.Gov*
NCT04482413	A Study to Evaluate the Safety and Efficacy of AstroStem in Treatment of Alzheimer’s Disease	Phase 2b, study with 2 treatment arms to compare efficacy and safety of AstroStem (autologous adipose tissue derived mesenchymal stem cells) vs. Donepezil treatment.	Active (estimated study completion date: December 2023)	United States	Nature Cell Co. Ltd.	*ClinicalTrials.Gov*
NCT02054208	Safety and Exploratory Efficacy Study of NEUROSTEM® Versus Placebo in Patients with Alzheimer’s Disease	Double-blind, single-center, phase I/IIa. The study will be divided into two stages: dose-escalation in stage 1 and randomized and multiple-dose cohort parallel design in stage 2. 45 patients with mild to moderate AD will be selected. The low-dose procedure will consist of 3 repeated intraventricular administration of 1×10^∧^7 human umbilical cord blood-derived mesenchymal stem cells (UCB-MSCs) cells/2 mL, via an Ommaya Reservoir, at 4-week intervals. High-dose procedure with 3×10^∧^7 UCB-MSCs cells/2 mL. Safety, dose-limiting toxicity, and efficacy will be accessed.	Completed (estimated study completion date: December 2019).	Korea	Medipost Co Ltd.	*ClinicalTrials.Gov*
NCT03117738	A Study to Evaluate the Safety and Efficacy of AstroStem in Treatment of Alzheimer’s Disease	A randomized, double-blind, placebo-controlled, parallel-group comparison study in subjects with AD to evaluate the safety and efficacy of Autologous adipose tissue-derived Mesenchymal Stem Cells (AdMSC). 21 subjects were randomized to receive either intravenous AdMSC or a placebo control (saline with 30% auto-serum). The procedure repeated 9 times at a 2-week interval.	Completed (estimated study completion date: June 2019)	United States	Nature Cell Co. Ltd.	*ClinicalTrials.Gov*
NCT01297218	Stereotactic brain injection of human umbilical cord blood mesenchymal stem cells in patients with Alzheimer’s disease dementia	Open-label, single-center, phase 1 clinical trial to evaluate the safety and dose-limiting toxicity of stereotactic brain injection of human umbilical cord blood–derived mesenchymal stem cells (hUCB-MSCs). The low-dose (*n* = 3) and high-dose (*n* = 6) groups received a total of 3.0×10^∧^6 cells/60*μ*L and 6.0×10^∧^6 cells/60*μ*L, respectively, into the bilateral hippocampus and right precuneus.	Completed (estimated study completion date: December 2011)	Korea	Medipost Co Ltd.	*ClinicalTrials.Gov*

There are several registered active clinical trials (Clinicaltrials.gov. https://clinicaltrials.gov Accessed June 2021) (Table 1). All these trials are using MSCs, either isolated from adipose tissue (NCT04388982 and NCT04228666), placenta (NCT02899091), umbilical cord blood (NCT02054208), bone marrow (NCT03724136 and NCT02600130), or are from an unspecified source (NCT02833792). Due to their unique properties, such as rapid proliferation, high differentiation capacity, and ability to migrate into the site of damage, MSCs are increasingly being used for cell therapy and tissue regeneration [107]. However, it remains to be established whether MSCs would differentiate only to neurons or to other cell types in a tissue with cellular heterogeneity. This is important because it is often assumed that transplanted SCs will give rise only to the cell lineage of interest. In these ongoing trials, the delivery routes for MSCs are different: intraventricular injection, intranasal, or intravenous administration. Intranasal administration is a promising route of delivery of SCs to the CNS that has emerged in recent years. Cells might cross the olfactory epithelium, pass into a space adjacent to the periosteum of the turbinate bones, and then enter the subarachnoid space [108]. One of the biggest obstacles in AD therapeutics is that most of the current drugs are introduced too late, according to the natural course of the disease. Therefore, new therapeutic approaches should target earlier stages in the AD progression before generalized neurodegeneration occurs and dementia has been established [109]. According to this hypothesis, an essential step in developing any SC therapy is to choose a presymptomatic or early AD stage and thus limit neurodegeneration and/or exert neuroprotective effects. Since later AD stages, the strategies that use iPSC should be more elaborate due to the generalized neurodegeneration process [110, 111]. More research and preclinical trials are still required to elucidate the mechanisms of action of SCs.

### Cell-derived exosomes as a possible alternative for stem cell therapy in AD

Notably, one of the ongoing studies (NCT04388982) includes the use of exosomes derived from adipose mesenchymal stem cells (MSC-Exos). Exosomes are produced by the endosomal network, and can transfer functional molecules like proteins, lipids, and RNA. It has been postulated that the transfer of damaged neuronal cell-derived exosomes could lead to the spread of AD. Besides, prion-like mechanisms involved in AD would include altered cell communication due to alterations of the endosomal/lysosomal secretion system. In that sense, exosomes isolated from SCs could promote neuroplasticity and neural replacement in AD [112]. MSC-Exos therapy has been suggested to be a potential alternative for AD treatment due to its immunomodulatory properties and promoting the Aβ degradation. Recently, in a study using AD transgenic mice, it was shown that MSC-exos could play a fundamental role in the Aβ degradation through regulating the activity of the enzymes neprilysin and the insulin-degrading enzyme [113, 114]. Likewise, in AD transgenic mice, it has been shown that MSC-Exos could have neuroimmunomodulatory effects by reducing neuroinflammation and improving memory and learning processes [115, 116]. MSC-Exos could have anti-inflammatory effects regulating the activation of microglia and astrocytes and the release of cytokines [117]. Therefore, it has been suggested that MSC-Exos-based therapy may have several benefits: high safety profile, anti-inflammatory effects, low immunogenicity, low risk of tumorigenesis, avoiding mutations and DNA damage caused by cell transplantation readily cross the BBB [118–122]. However, this therapy still has several limitations and challenges: 1) MSC-Exos storage, isolation and standardization studies are required for better comparability and reproducibility; 2) it is still difficult to establish the specific source of MSC-Exos with greater therapeutic potentials; 3) MSCs can secrete harmful cytokines and have paracrine effects; 4) the adverse effects, the optimal dose and route of administration are not yet clarified [123, 124].

### The link between 3D printing technology and stem cells

The techniques based on bioprinting and the implementation of 3D organoids are beneficial tools for developing innovative cell cultures. They generate 3D models where cells can be eliminated systematically and control their growth in similar structures to tissues [125]. In this way, the association of 3D bioprinting technologies and those based on iPSCs could develop more reliable and realistic cell cultures and, above all, focus the investigation of organoids derived from differentiated cells of patients. Generally, 3D human brain cultures are generated through iPSC differentiation, either in neuronal cell aggregates or even more complex brain organoids. The first one was generated from human neuronal progenitors cultured in a 3D suspension, while the latter are generally obtained through serum-free floating cultures of embryoid body-like aggregates [126]. Based on this last method, a new class of organoids called “mini-brains” has been obtained, presenting different discrete but interdependent brain areas [127]. Brain organoids have been used to study, analyze, and implement new therapeutic approaches for neurodegenerative diseases [126, 128]. Recently, a self-organizing 3D human array was developed from iPSCs derived from AD patients with a duplication in the *APP* gene. This model mimicked the main AD hallmarks, such as pathological Aβ aggregates and tau phosphorylation. It also allowed the implementation of a therapeutic strategy focused on β- and *γ*-secretase inhibitors [129]. However, the organoid-based technology presents some limitations [130]: 1) they present only an epithelial monolayer without a tissue microenvironment, like the one present in the CNS; 2) the proper maturation of the organs or tissues has not yet been achieved; 3) the dependence of the extracellular matrix “Matrigel”, which is derived from mouse tumor lines and could facilitate tumor formation and impede drug penetration; 4) Molecular inhibitors and/or growth factors in the cell culture medium could generate effects on the responses of organoids to drugs. Future research is required to evaluate these limitations.

## LIMITATIONS OF SCS THERAPY IN ALZHEIMER’S DISEASE

In recent years, SCs therapies have offered the potential to treat diseases with no current successful therapies. Sometimes called “master cells”, SCs have the potential to repair, restore, replace, and regenerate cells. However, the molecular mechanism by which SCs may rescue the disease has not been defined [131]. Despite some early promise, there are very few reports of completed registered trials involving SCs transplantation in AD patients. Furthermore, studies conducted with animal models have failed to provide evidence of either efficacy or safety.

There are social and ethical concerns about using SC-based technology, which also limits federal funds and therefore advances in research. FDA is concerned that some clinics may be inappropriately conducting clinical SCs treatment without FDA review to ensure that it is reasonably safe and effective [132]. The only SC-based products that are FDA-approved for use in the United States consist of blood-forming SCs (hematopoietic progenitor cells) derived from cord blood. These products are approved for limited use in patients with disorders that affect the body system involved in the production of blood [133]. Despite not having conclusive results about the safety and efficacy of SCs, many clinics are applying therapies to patients affected by AD. The type of cells they use, dose, form, and duration of treatment, among other variables, can vary. In the United States, between 2009 and 2014, clinics with websites that carried out SCs treatments overgrew, at least doubling every year [134]. By 2017, there were more than 700 companies [135]. To this number, we must add other non-regulated clinics or laboratories, which may operate without a well-defined regulatory framework. The cost of SCs therapy can range from USD $5,000–$50,000. This price can vary significantly depending on the number and origin of the SCs, viability, country, and whether the clinic or lab is regulated [136]. Other costs, such as pre-surgical lab work, medications, or hospital stay, should also be considered. In the European Union, specific rules (Regulation (EC) No 1394/2007) were introduced in 2007 to ensure that SCs therapy is subject to appropriate authorization and control, with quality standards for the traceability of materials, treatment protocols, and patient follow-up measures. On the other hand, Japan, which has invested billions of yen in PSCs, made by reprogramming an individual’s adult cells and that can develop into any body tissue, have created a fast-track system for SCs treatments and regenerative medicine [137]. The use of SCs also poses a source of ethical conflicts. The ESCs, the most common pluripotent SCs, can be removed from human embryos, potentially becoming a human. Because of this, scientists have focused on isolating SCs without endangering the embryo [55].

The use of SCs can lead to side effects observed in clinical practice. One of the major concerns is tumorigenicity. Self-renewal and plasticity are properties that characterize cancer cells, and SCs transplantation could prepare fertile ground for tumor development. It has been hypothesized that SCs can even mimic tumor development due to high diversity of cells [138]. Also, isolating SCs from their original niche, which provides them a specific molecular regulator and a suitable physicochemical environment, could have unexpected or undesirable outcomes once integrated into different organs of the body [131].

Several cases of immune rejection or graft versus host disease have been reported. Although most clinics claim to use SCs from the patient’s body fat, bone marrow, and blood, on other occasions, cells from amniotic fluid, placental tissue, umbilical cord tissue, and even unknown sources of cells from different donors are used. Due to the risk of rejection and achieving graft survival, immunosuppressive therapy has been suggested, promoting MHC class I-dependent NK cell-mediated elimination [139]. Nevertheless, immunosuppressive therapy could favor opportunistic infections in patients. Therefore, for the advancement in SCs-based therapies, it becomes essential to establish the mechanisms by which the host tolerance to the donor graft is regulated. Interestingly, the use of gene editing has been proposed to avoid immune rejection. Through this technology, the SCs genome can be manipulated *ex vivo* to correct the underlying genetic defect before transplantation and prevent the host’s immune response [140, 141].

Therefore, at present, it is probably best to use the patient’s cells and devolve them into their pluripotent stage of development. Genomic instability is also recognized as one of the most critical hurdles in the field of SCs based therapy [142]. In addition to the above, the FDA includes many safety concerns: administration site reactions, the ability of cells to move from placement sites, and change into inappropriate cell types or multiply and failure of cells to work as expected.

In the case of AD, the therapy with SCs generates many unanswered questions. There are no data on what type of SCs would be the most effective for this treatment: differentiated or undifferentiated pluripotent. Likewise, information about cell concentration, number of effective doses, and duration of treatment is missing. The type of transplantation that should be carried out is not defined: localized (by stereotactic injections) or peripheral; and how cell migration to the affected brain area occurs. There is no evidence on the effectiveness of SCs therapy in humans: how SCs can eliminate the inflammatory and toxic environment generated by the Aβ peptide; how connections between the healthy cells would be originated; or how the cell repopulation would affect other metabolic pathways. Whether SCs transplantation would prevent communication between damaged cells or the spreading of pathological tau protein between neurons [23, 24, 71, 72] remains elusive. Finally, since it has not been possible to demonstrate the safety of this type of therapy for AD, continuous monitoring of the patient would be required to avoid both short and long-term harmful side effects.

### Alternatives to overcome the limitations of stem cell-based therapy

As we have described, most of the current research focuses on therapy based on the transplantation of externally cultured cells. However, they have several limitations, including rejection and post-transplant immune response. Therefore, it has been proposed that the use of *in vivo* reprogramming directly may be an alternative to overcome these obstacles. *In vivo* reprogramming consists of using internal cells to regenerate specific cells and restore tissues. In this sense, viral injections can be used, which ectopically express transcription factors in a particular type of cell, which would be reprogrammed in a different kind of target cell. This technology has been used to regenerate new neurons both at the brain and spinal level.

The transcription factor called Neurogenin2 (Ngn2) has shown efficacy in the direct *in vivo* reprogramming of astrocytes into functional neurons in adult mouse brains. However, the reprogramming efficiency is low [143, 144]. Other studies have shown that the reprogramming efficiency increases when Ngn2 is co-expressed with Bcl2 [143]. Likewise, a high reprogramming efficiency mediated by neuronal differentiation 1 (NeuroD1) has been demonstrated. Previous studies showed in brain slices that neuron converted with NeuroD1 matured after 1 month of treatment and also created new connections with pre-existing neurons [145]. Interestingly, it has been found that direct reprogramming in the mouse brain can be achieved by combining the transcription factors Ascl1, Brn2, and Myt1l, converting fibroblast cells into neurons astrocytes into neuron-like cells [146].

The Neuron-glial antigen 2 (NG2), which is also called “oligodendrocyte precursor cells”, can also differentiate into neurons according to experiments carried out in the adult mouse brain. These studies showed that NeuroD1 also reprograms NG2 cells into glutamatergic and GABAergic neurons [145]. Future research is required to advance clinical trials. One of the main challenges in SCs therapy and *in vivo* reprogramming therapy is neuronal viability, which could be interfered with by a neurodegenerative disease, such as AD. Since there is a neuroinflammatory environment triggered by glial activation [147] in AD, newly differentiated neurons may not survive and may not create new synaptic connections. Therefore, it is necessary to establish complementary therapeutic strategies that favor the survival of newly differentiated neurons. Besides, since most neurological diseases preferentially damage specific neural subtypes, it is required to advance in this technology to generate these particular subtypes. To date, the formation of new neuronal networks and functional connectivity remains a challenge.

## CONCLUSION

Many SCs therapies are still experimental. Although SCs products may offer the enormous potential to treat many medical diseases, there is not enough scientific evidence to ensure that its use is safe and provides some health benefits. AD is a complex disease with devastating effects for both patients and families, so finding an effective treatment has become an urgent need. Although several studies with SCs in animal models for AD have been carried out, the difference in the molecular patterns of these models to humans, together with the contradictory findings obtained, have prevented the translation of this technique to the treatment of AD. More profound knowledge of the cellular mechanisms related to the use of SCs, in both animal models and humans is essential before the benefits of such therapy can be established in the clinic.
